# Prediction of the importance of auxiliary traits using computational intelligence and machine learning: A simulation study

**DOI:** 10.1371/journal.pone.0257213

**Published:** 2021-11-29

**Authors:** Antônio Carlos da Silva Júnior, Michele Jorge da Silva, Cosme Damião Cruz, Isabela de Castro Sant’Anna, Gabi Nunes Silva, Moysés Nascimento, Camila Ferreira Azevedo

**Affiliations:** 1 Department of General Biology, Federal University of Viçosa, Viçosa, Minas Gerais, Brazil; 2 Centro de Seringueira e Sistemas Agroflorestais, Instituto Agronômico (IAC), São Paulo, Brazil; 3 Department of Mathematics and Statistics Scholar, R. Rio Amazonas, Ji-Paraná, RO, Brazil; 4 Department of Statistics, Federal University of Viçosa, Viçosa, Minas Gerais, Brazil; KGUT: Graduate University of Advanced Technology, ISLAMIC REPUBLIC OF IRAN

## Abstract

The present study evaluated the importance of auxiliary traits of a principal trait based on phenotypic information and previously known genetic structure using computational intelligence and machine learning to develop predictive tools for plant breeding. Data of an F_2_ population represented by 500 individuals, obtained from a cross between contrasting homozygous parents, were simulated. Phenotypic traits were simulated based on previously established means and heritability estimates (30%, 50%, and 80%); traits were distributed in a genome with 10 linkage groups, considering two alleles per marker. Four different scenarios were considered. For the principal trait, heritability was 50%, and 40 control loci were distributed in five linkage groups. Another phenotypic control trait with the same complexity as the principal trait but without any genetic relationship with it and without pleiotropy or a factorial link between the control loci for both traits was simulated. These traits shared a large number of control loci with the principal trait, but could be distinguished by the differential action of the environment on them, as reflected in heritability estimates (30%, 50%, and 80%). The coefficient of determination were considered to evaluate the proposed methodologies. Multiple regression, computational intelligence, and machine learning were used to predict the importance of the tested traits. Computational intelligence and machine learning were superior in extracting nonlinear information from model inputs and quantifying the relative contributions of phenotypic traits. The *R*^2^ values ranged from 44.0% - 83.0% and 79.0% - 94.0%, for computational intelligence and machine learning, respectively. In conclusion, the relative contributions of auxiliary traits in different scenarios in plant breeding programs can be efficiently predicted using computational intelligence and machine learning.

## Introduction

Plant breeding is effective for increasing crop productivity. Its main objective is to increase the frequency of desirable alleles in plant populations to develop superior crops with high productivity, disease and pest resistance, abiotic stress tolerance, and environmental adaptability [1, 2]. Quantifying the importance of traits allows the breeder to determine strategies for increasing selection efficiency (e.g., indirect selection), to perform extensive phenotypic evaluation of the germplasm, and to predict the future performance of traits with low heritability (*h*^2^) and/or difficulties in measurement.

The efficiency of selection can be increased by selecting secondary traits that are easy to measure, have high *h*^2^, and are closely correlated with the principal trait. Indirect selection through a secondary trait may be more efficient than direct selection if *h*^2^ is higher for the secondary trait than for the primary trait, and if the genetic correlation between the primary and secondary traits is sufficiently strong [3].

Although the simultaneous evaluation of traits in a plant breeding program provides massive data, identifying the most important predictive phenotypic trait is a challenge for breeders. Conventional methods of phenotypic trait selection are based on multiple linear regressions. For instance, mixed models are often used in plant breeding programs [4]. In principle, these models evaluate the relationship between a dependent phenotypic trait and two or more independent phenotypic traits [6]. However, this method has the ability to analyze multidimensional data and cannot capture complex, multivariate relationships among phenotypic traits [5–7].

In this context, computational intelligence may serve as an alternative to predict complex traits from auxiliary traits [2, 8–11]. Artificial neural networks (ANNs) are highly parameterized nonlinear models with sets of processing units called neurons, and they can be used to predict the relationships between the input and output signals of a complex system [12]. ANNs are powerful prediction tools compared with conventional models, such as linear regression [13–15]. In addition, these networks can reproduce the importance of each predictive trait, rendering it easily interpretable [16]. Despite their overall high predictive performance, ANNs have been neglected in studies of the importance of traits.

Multilayer perceptron (MLP) and radial basis function (RBF) networks are the most commonly used ANNs. The MLP classifier has the typical architecture of an ANN with at least one hidden layer and one output layer, both with nonlinear and differentiable transfer functions [17–20]. The RBF network has a simpler structure and a faster learning algorithm than other ANNs [21, 22]. It comprises three layers: an input layer, a hidden layer, and an output layer. A large amount of nonlinear information is accepted by the input layer and then transmitted through the hidden layer. Finally, the results are obtained from the output layer [21]. Thus, RBF networks have been successfully used in genomic selection [23–27] and variable selection [2, 28].

Other interesting alternatives for predicting and quantifying the importance of auxiliary traits are machine learning-based methodologies, such as decision trees [2, 7, 19] and their refinements, such as bagging, random forest, and boosting [2, 29]. These methodologies allow breeders to predict the importance of traits using measures based on the Gini coefficient and entropy index [30]. Computational intelligence, machine learning, and multiple regression have proven to be efficient predictive tools for various agricultural crops. For instance, in soybean, phenotypic characteristics related to seed yield prediction (row spacing and seeding density) were studied using these methods [7]. Moreover, [5] applied these methodologies to compare and predict pest population dynamics based on the climatic and phenological factors of the host plant.

To this end, the present study evaluated the importance of auxiliary traits of a principal trait based on phenotypic information and previously known genetic structure using computational intelligence and machine learning to develop predictive tools useful in plant breeding programs.

## Material and methods

### Dataset

A set of simulated data of an F_2_ population represented by 500 individuals, derived from a cross between contrasting homozygous parents, was used.

### Phenotyping

Eleven phenotypic traits (PTs) were simulated using previously established means and *h*^2^ estimates. The *h*^2^ values used were 30%, 50%, and 80%, respectively (Table 1). The traits were established by the action of 40 allele loci based on 1,000 markers in 10 linkage groups (LGs) with differential additive effects. Previous simulation studies used fewer than 20 quantitative trait loci (QTLs) [23, 24, 31]; therefore, we explored any number of QTLs in the present study. The weights of importance of the loci on the total genotypic variability of the traits were established from a uniform distribution.

**Table 1:** Description of phenotypic traits (PT) in relation to heritability (*h*^2^) and the distribution of linkage groups (LG).

PT	*h* ^2^	LG1	LG2	LG3	LG4	LG5	LG6	LG7	LG8	LG9	LG10
1	0.5	8	8	8	8	8	-	-	-	-	-
2	0.5	-	-	-	-	-	8	8	8	8	8
3	0.3	4	4	4	4	4	4	4	4	4	4
4	0.5	4	4	4	4	4	4	4	4	4	4
5	0.8	4	4	4	4	4	4	4	4	4	4
6	0.3	4	4	4	-	-	8	8	4	4	4
7	0.5	4	4	4	-	-	8	8	4	4	4
8	0.8	4	4	4	-	-	8	8	4	4	4
9	0.3	4	-	-	-	-	8	8	8	8	4
10	0.5	4	-	-	-	-	8	8	8	8	4
11	0.8	4	-	-	-	-	8	8	8	8	4

Phenotypic traits (PT) were simulated using previously established means and heritability (*h*^2^). The *h*^2^ values used were 30%, 50%, and 80%. The traits were established by the action of 40 locus alleles based on 1,000 markers in 10 LGs with differential additive effects.

PT1 was used as the principal trait of prediction with 50% heritability and 40 control loci of the trait distributed in LGs 1, 2, 3, 4, and 5 (Table 1). Ten auxiliary traits with known genetic control loci were also considered. PT2 was simulated assuming the same complexity as PT1 but without any genetic relationship to PT1 and without pleiotropy or a factorial link between the control loci of PT1 and PT2. Thus, we hypothesized that PT2 is the least important trait for prediction.

PT3, PT4, and PT5 in different scenarios represented other important traits (Table 1); they shared a large number of controlling loci with PT1 but could be distinguished by the differential action of the environment on them, as reflected in the estimates of heritability. Similarly, subsets of auxiliary traits, namely PT6, PT7, and PT8, as well as PT9, PT10, and PT11, with decreasing importance, were tested. The following statistical model was used:

$$Y_{i}=\mu +G_{i}+\varepsilon_{i}$$ (1)

where Y_i_ is the simulated observation of the trait of the i^th^ individual, μ is the general average of the trait, whose value is specified by the breeder, G_i_ is the effect associated with the i^th^ individual, with $G_{i}∼N(0,\sigma_{g}^{2})$; ε_i_ is the random error, where ε_i_~N(0, σ^2^), and $\sigma^{2}=(1-h^{2})\sigma_{g}^{2}/h^{2}$.

The total phenotypic value in the epistatic model, expressed by an individual belonging to the population, was estimated using the following equation:

$$Y_{i}=\mu +\sum_{j=1}^{40}p_{j}\alpha_{j}+\sum_{j=1}^{39}p_{j}\alpha_{j}\alpha_{j+1}+\varepsilon_{i}$$ (2)

here, *α*_*j*_ = *a*_*i*_+*d*_*i*_ and $\frac{a_{i}}{d_{i}}$ = gmd, where *μ*+*a*_*j*_, *μ*+*d*_*j*_, and (*μ*−*a*_*j*_) are the genotypic values associated with classes AA, Aa, and aa, assumed to be equal to 1, 0, and -1, respectively, when coded; *p*_*j*_ is the contribution of the locus j to the expression of the trait, with a uniform distribution; and *d*_*i*_ is average degree of dominance of each trait (*d*_*i*_ = 0.5).

### Genotyping

A total of 1,000 codominant molecular markers, with two alleles per marker, were used. These markers were distributed in a genome established by 10 LGs, reflecting a diploid species with 2n = 2x = 20. Each LG was 100 centimorgans; thus, 100 markers were evenly spaced. All the markers are described in Table 2.

**Table 2:** Location of markers and control loci of traits.

Linkage group	Markers	Control loci
1	1–100	10 20 30 40 50 60 70 80
2	101–200	110 120 130 140 150 160 170 180
3	201–300	210 220 230 240 250 260 270 280
4	301–400	310 320 330 340 350 360 370 380
5	401–500	410 420 430 440 450 460 470 480
6	501–600	510 520 530 540 550 560 570 580
7	601–700	610 620 630 640 650 660 670 680
8	701–800	710 720 730 740 750 760 770 780
9	801–900	810 820 830 840 850 860 870 880
10	900–1000	910 920 930 940 950 960 970 980

Eight control loci were used for each link group.

Four different scenarios were considered to predict phenotypic traits (PT1). These scenarios differed in terms of the four loci controlling quantitative traits PT3 to PT11 (Table 2). Scenarios 1 (10 20 30 40) and 2 (50 60 70 80) represented the first four and the last four loci controlling the quantitative traits, respectively (Table 2). Scenario 3 (i.e., 10 20 70 80) represented the first two and last two loci controlling quantitative traits. Finally, scenario 4 (30 40 50 60) represented the central loci controlling the quantitative traits (i.e., the first and last two loci were excluded).

### Indirect selection gain through principal trait

Indirect selection gain through principal traits was estimated as described by [32] using the following equation:

$${GS}_{y(x)}=ih_{x}r_{g}s_{gy}$$

Where *GS*_*y*(*x*)_ is the indirect selection gain in y (x), which is selection intensity (0.9659); x is the principal trait under selection; *h*_*x*_ is the square root of heritability; $r_{g}=\frac{h_{x}}{h_{y}}$ is the absolute value of the estimated genetic correlation (estimated from genetic covariances) between the principal traits x and y; and *s*_*gy*_ is the phenotypic standard deviation.

### Prediction of the importance of phenotypic traits

To predict PT1 and determine the importance of other traits (PT2 to PT11), multiple regression, computational intelligence, and machine learning were used.

#### Stepwise multiple regression

Stepwise multiple regression selects a predictor trait at the expense of the coefficient of determination (*R*^2^) between the dependent and independent traits. For prediction using stepwise multiple regression, PT1 was used as the principal trait and the others as auxiliary traits. The *R*^2^ values were used to verify the extent to which the independent traits explained the total variation in the dependent trait. The following model was used to predict PT1:

$$y=\beta_{0}+\beta_{1}x_{1}+\beta_{2}x_{2}+\cdots +\beta_{k}x_{k}+\varepsilon$$ (3)

Where *y* is the response variable (PT1); *x*_1_ to *x*_*k*_ (PT2 to PT11) are the explanatory variables; *β*_0_ represents the intercept; *β*_1_ and *β*_*k*_ are the coefficients associated with the variables *x*_1_ to *x*_*k*_; and *ε* is the residual effect.

*R*^2^ was calculated using the following equation:

$$R^{2}=1-\frac{\sum_{i=1}^{n}{\left(y_{i}-{\hat{y}}_{i}\right)}^{2}}{\sum_{i=1}^{n}{\left(y_{i}-{\bar{y}}_{i}\right)}^{2}}$$ (4)

where the actual values are indicated by *y* and predicted values by $\hat{y}$.

Pearson’s correlation analysis was used to evaluate the relationship between PT1 and other traits [33]. The first and second groups constituted the phenotypic traits PT1 and PT2, respectively. According to the number of shared control loci with the principal trait, the third group was composed of PT3, PT4, and PT5; the fourth group comprised PT6, PT7, and PT8; and the fifth group comprised PT9, PT10, and PT11.

### Computational intelligence

MLP and RBF neural networks were used for information processing and prediction of the importance of phenotypic traits, as described below.

#### MLP

The MLP networks are characterized by having at least one intermediate (hidden) layer located between the input and output layers. For the best efficiency of this network, before training, the data were normalized by an interval between − -1 and 1. The maximum number of training periods was set to 5,000, and the minimum mean square error (MSE), to stop processing the network, was set at 1.0 × 10^−3^. All trained networks included one neuron in the output layer and a single hidden layer with 30 neurons. The *sigmoid tangent* activation function was used in the hidden layer, and Bayesian regulation backpropagation was used *as the training algorithm*.

To quantify the importance of phenotypic traits using the MLP network, two methodologies were used, namely Garson’s algorithm (1991) and modified by Goh [34]. In this approach, the neural network connection weights are partitioned to determine the relative importance of each input variable in the network. This function was implemented using the method described by [34]. In this method, the relative importance of each variable was determined as the absolute magnitude. For each input node, all weights connecting an input through the hidden layer to the response variable are identified to return a list of all weights specific to each input variable. In the second methodology, the importance of traits (inputs) was evaluated through the impact of de-structuring or disturbance of information on a given input on the *R*^2^ estimates. This importance was estimated by exchanging information on or making the phenotypic value of each trait constant and verifying the changes in the *R*^2^ estimates. When the values of a trait are disturbed, the value of *R*^2^ decreases, indicating that the trait is important relative to the others for the purpose of prediction.

The relative importance of the variable, measured by the reduction in R^2^ and obtained by permuting its values, was quantified using the following equation:

$${pVR}_{x_{i}}=R_{obs}^{2}-{\bar{R}}_{perm,x_{i}}^{2}$$ (5)

where $R_{obs}^{2}$ is the *R*^2^ of the ANN topology obtained using the original predictor variables and established by the square of the correlation between the predicted and observed values, and $R_{perm,x_{i}}^{2}$ is the *R*^2^ of the same topology as the ANN and was obtained from a dataset in which the values of *x*_*i*_ were altered by the permutation procedure.

#### RBF

RBF network is characterized by having only one hidden layer and using a Gaussian activation function. The structure of the RBF that best predicted PT1 was established with 10 to 30 neurons (increased by 2 at each processing step) and a radius of 5 to 15 (increased by 0.5, at each processing step). The efficiency of prediction was measured based on *R*^2^, and the relative importance of each trait was measured by de-structuring the information on each explanatory phenotypic trait, as described for MLP above.

### Machine learning

Machine learning is one of the techniques used in artificial intelligence, and it allows for the detection of patterns in large datasets and the development of predictive models [35]. Learning algorithms based on decision trees are considered one of the most efficient and most used methods of supervised learning [36] to build predictive models of high precision, stability, and ease of interpretation. To predict PT1 and determine the importance of phenotypic traits through machine learning, decision trees with bagging, random forest, and boosting refinements were used. The quality of the predictive model fit was determined based on *R*^2^, and the MSE was used to quantify the importance of phenotypic traits.

The importance of the explanatory trait was determined by estimating the percent increase in MSE (%IMSE). %IMSE was derived for each predictor variable from the difference in MSE between the predictive measure based on the original dataset and that based on a permuted dataset, where the predictor in question was randomized [37]. To improve the predictive efficiency for the importance of traits, 5,000 trees were generated.

The relative importance of the variable (IV) was obtained by permuting its values and quantified using the following equation:

$${IV}_{x_{i}}={MSE}_{perm,x_{i}}-MSE,$$ (6)

where ${MSE}_{perm,x_{i}}$ is the mean quadratic error of the methodology obtained from the use of a dataset in which the *x*_*i*_ values were changed by the permutation procedure. *MSE* is the mean square error of the same methodology obtained from the original predictor variables and is a function of the square of the deviations between the predicted and observed values.

### Training and validation sets

The dataset was divided into two parts: a training and validation set. The training set included the same individuals for modeling using all methodologies and was composed of 80% individuals in each class, selected at random. The remaining 20% of individuals constituted the validation set. In previous studies, 60% to 90% of individuals constituted the training set [26, 31]. For the training and validation of the algorithms used, cross-validation (k-fold) was performed with k = 10 partitions [38].

Data simulation and analysis were performed using the R package NeuralNetTools [15] and Genes [39].

## Results and discussion

### Summary of key findings

The *R*^2^ estimates for all methodologies, using the explanatory traits for PT1, are shown in Table 3. Based on these results, the methodologies used were compared and defined, proving the most efficient approach to PT1 prediction. Higher *R*^2^ values indicate that the principal trait of prediction is more adaptable than the other explanatory phenotypic traits [2, 40].

**Table 3:** Maximum estimate of the coefficient of determination (*R*^2^) for all methodologies using the explanatory traits for phenotypic trait 1.

Scenario	CI		ML				MR
	MLP (NN)	RBF	DT	RF	BA	BO	Stepwise
1	83.02 (30)	54.42	51.00	94.40	94.64	82.12	41.03
2	77.89 (29)	48.51	49.24	93.82	93.83	79.74	33.88
3	75.49 (29)	44.04	43.66	93.99	93.89	79.86	34.82
4	82.14 (25)	47.06	45.75	93.49	93.32	80.01	38.16

CI: computational intelligence, ML: machine learning, MR: multiple regression, MLP: multilayer perceptron, RBF: radial basis function, DT: decision tree, RF: random forest, BA: bagging, BO: boosting. NN: number of neurons in hidden layer.

Stepwise multiple regression provided the lowest estimate of *R*^2^ (Table 3), indicating the existence of non-linear associations among the explanatory phenotypic traits not considered in the model; in the present study, this result can be attributed to the epistatic action between the control loci of each trait. In multiple linear regression, the absolute value of the *t-statistic* is commonly used as a measure of variable importance. Computational intelligence and machine learning were superior in extracting nonlinear information from model inputs (Table 3). However, in all scenarios, *R*^2^ was lower for computational intelligence than for machine learning (Table 3).

The *R*^2^ values were 83.03%, 77.89%, 75.49%, and 82.14% for scenarios 1, 2, 3, and 4, respectively, in the MLP network with only one neuron in the output layer and a single hidden layer (Table 3). The differences in results obtained with different methodologies indicate that the scenarios influenced the estimation of *R*^2^ and, consequently, the prediction of PT1. Similar results have been reported in studies predicting corn and soybean yield based on climatic conditions using ANNs (*R*^2^ = 0.77 for corn, and 0.81 for soybean) and multiple linear regression (*R*^2^ = 0.42, maize and 0.46 for soybean, respectively) [41]; moreover, the development of linear regression models was time-consuming, and ANN models were superior in terms of accurately predicting corn and soybean yields under typical climatic conditions. Silva et al. [9] applied ANNs to simulated traits with 40% and 70% heritability for predicting genetic values and gains and found more efficient selection using ANNs than using maximum likelihood (genotypic mean). In addition, several studies have used this parameter (*R*^2^) to verify the effectiveness of methodologies for the prediction or classification of simulated populations [2, 9, 28, 42, 43].

Twenty-five neurons were established for the MLP network, and an *R*^2^ of 82.14% was obtained for scenario 4. For scenarios 2 and 3, 29 neurons were established, and *R*^2^ values of 77.89% and 75.49% were obtained, respectively. In scenario 1, 30 neurons were established, and a maximum *R*^2^ value of 83.02% was obtained (Table 3). High (>90%) *R*^2^ estimates for all analyses were obtained using machine learning with random forest and bagging refinements (Table 3). With boosting, these estimates were >80%, indicating an efficient estimate of *R*^2^. Decision trees were not superior to the remaining machine learning methods, and the *R*^2^ values with decision trees were similar to those with RBF and stepwise multiple regression.

Thus, machine learning is indeed more efficient for the selection of phenotypic traits because it can deal with reduced or redundant information on phenotypic traits [44]. Costa et al. [45] assessed the importance of variables by bagging, random forest, boosting, decision tree, PML, and RBF and reported that PML and RBF achieved better results. After verifying the efficiency of different computational intelligence and machine learning methodologies in predicting PT1, we sought to identify the explanatory phenotypic traits that should be prioritized and established as auxiliary traits for indirect selection, as described below.

### Importance of phenotypic traits by Pearson’s correlation

Pearson’s correlation coefficients were calculated between PT1 and other phenotypic traits in the four scenarios (Table 4).

**Table 4:** Pearson’s correlation coefficients between phenotypic trait (PT) 1 and other traits in the four scenarios.

PT	Scenario			
	1	2	3	4
2	0.08^ns^	0.10*	-0.08^ns^	-0.05^ns^
3	0.07^ns^	0.38**	0.34**	0.27**
4	0.43**	0.30**	0.38**	0.47**
5	0.47**	0.43**	0.39**	0.47**
6	0.14**	0.05^ns^	0.05^ns^	0.04^ns^
7	0.25**	0.19**	0.26**	0.14**
8	0.12**	0.24**	0.15**	0.23**
9	0.03^ns^	-0.13**	0.06^ns^	0.21**
10	0.03^ns^	-0.04^ns^	0.03^ns^	0.12**
11	0.04^ns^	0.03^ns^	0.02^ns^	0.08^ns^

Significant at **1% and *5% probability of error by t-test. ns: non-significant. Scenario 1 represents the first four control loci, scenario 2 represents the last four control loci, scenario 3 represents the first two and last two control loci, and scenario 4 represents the central control loci (excluding the first and last two loci).

PT1 showed a positive and significant correlation with PT5 (P < 0.01) in all scenarios, probably because of the large number of shared control loci and high *h*^2^ (80%) of PT5. Meanwhile, the correlation between PT1 and PT2 was not significant (P > 0.05) in all scenarios, except in scenario 2, which is consistent with the simulation results and the absence of shared control loci between these two traits. These results were expected because PT2 had no genetic relationship with PT1 due to the absence of pleiotropy or a factorial link between the loci controlling these phenotypic traits.

**Fig 1:**
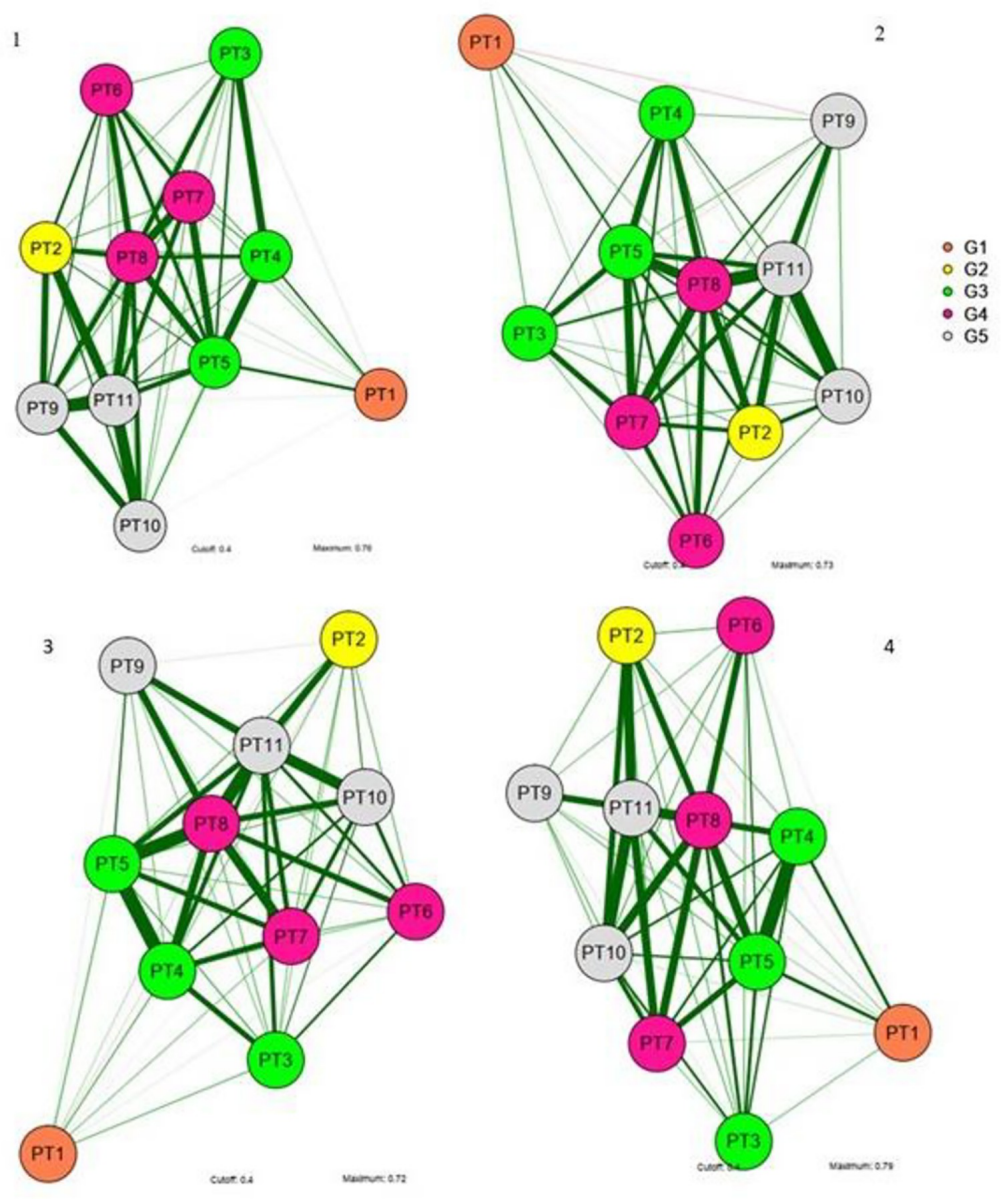


The phenotypic correlation network is shown in Fig 1. Consistent with the results in Table 4, these correlations were considered stronger relative to those among the groups. Group 2, represented by PT2, was positioned far from the principal trait (PT1) because of the lack of shared loci or genetic relationships. Group 3, represented by PT3, PT4, and PT5, was closer to PT1 and represented the most important traits for predicting PT1, particularly PT5, which had the highest *h*^2^ value. The placement of groups 4 and 5 relative to PT1 was consistent with the simulation of genetic structures, allowing satisfactory prediction of importance, albeit lower than expected relative to group 3 but higher than expected relative to PT2.

In breeding programs, understanding the meaning and degree of association between traits plays an important role in the development of selection strategies that facilitate the production of superior genotypes. One of the most used techniques to estimate these associations is the Pearson correlation, which is interpreted as the strength of the linear association between a pair of characteristics [46]. When more than two characteristics are considered, this measure alone does not show the real meaning and magnitude of the interrelations, making it impossible to determine whether the associations are cause or effect [47]. Therefore, a path analysis is used when there are dependent (interest) characteristics and other characteristics whose interrelationships are of interest to the researcher [48]. This technique has been shown to be very useful for revealing cause-and-effect associations and providing support in indirect selection.

The estimates of the correlation coefficients can help improve our understanding of a complex character, such as production, but they do not determine the relative importance of the direct and indirect influences of the other characters on production [48]. This is because the correlation between two characteristics measures the association between both, but it does not determine the cause-and-effect relationship between them, which can be determined through the trail analysis [3]. When the correlation between explanatory characteristics increases, the difficulty in assessing their relative importance in predicting the dependent characteristic is greater [49, 50]. Machine learning and computational intelligence are approaches that, even in the presence of an association between explanatory characteristics and a high degree of multicollinearity, are able to make appropriate predictions and classifications.

### Indirect selection through principal traits

Table 5 shows percent indirect selection gains between PT1 and the other traits in the four scenarios. The highest percent indirect gain was achieved with PT5 (*h*^2^ = 80%) in all scenarios. In scenario 2, the highest indirect selection gain was achieved with PT3 and PT4 (3.94 and 2.12, corresponding to *h*^2^ = 30% and 50%, respectively). Meanwhile, in scenarios 3 and 4, the highest selection gain was achieved with PT4 (*h*^2^ = 50). Therefore, a greater number of shared control loci affected indirect selection gain. The success of indirect selection depends mainly on heritability and genetic correlation between the primary and secondary traits [32]; therefore, traits closely correlated traits with a greater heritability than the target trait have great potential for indirect selection.

**Table 5:** Percentage gain of indirect selection between phenotypic trait (PT) 1 and the other traits in the four scenarios.

PT	1	2	3	4
2	0.06	1.32	**-0.06**	**-1.07**
3	0.05	3.94+	0.25	1.97
4	0.31+	2.12+	0.28+	3.46+
**5**	0.34+	2.73+	0.29+	3.12+
6	0.1	-0.32	0.04	-0.27
7	0.18	1.05	0.19	1.18
8	0.08	1.63	0.11	1.26
9	0.02	-1.4	0.04	2.51
10	0.02	-0.42	0.02	0.76
11	0.03	0.06	0.02	0.32

+: minor importance in PT1 prediction Scenario 1 represents the first four control loci, scenario 2 represents the last four control loci, scenario 3 represents the first two and last two control loci, and scenario 4 represents the central control loci (excluding the first and last two loci).

### Importance of phenotypic traits by computational intelligence

#### MLP

The results of quantification of the importance of phenotypic traits using MLP after the permutation of traits and assignment of a zero value to the input trait are shown in Table 6. If *R*^2^ shows a great reduction after disturbing the values of a trait, the phenotypic trait is important relative to others for prediction. The relative importance of phenotypic traits based on the reduction in *R*^2^ (Table 6), independent of the heritability of each phenotypic trait and LGs, differed across scenarios. Permutation was efficient in quantifying the importance of PT5, which was the most important trait in all scenarios except scenario 4, in which PT4 was the most important trait (Table 6). The *R*^2^ values were 83.02%, 77.89%, 75.49%, and 82.14% in scenarios 1, 2, 3, and 4, respectively (Table 3). Furthermore, permutation was efficient in demonstrating PT2 as the least important trait in scenarios 3 and 4, which was expected based on the number of markers influencing the principal trait (PT1).

**Table 6:** Estimation of the coefficient of determination (*R*^2^) for the prediction of phenotypic trait 1 (PT1) using multilayer perceptron (MLP).

PT	Zero				Permutation			
	1	2	3	4	1	2	3	4
2	34.13	16.28	17.71	6.69	45.54	36.33	43.89*	44.14*
3	11.33	0.81	11.23	1.47	36.14	29.23	38.78	35.15
4	10.32	3.62	2.72	0.13	19.45	32.84	41.32	12.03+
5	11.93	1.22+	3.30	0.76	17.83+	18.72+	19.09+	16.29
6	14.52	17.54*	14.86	0.03+	50.35*	31.42	33.34	21.11
7	8.34	3.80	23.53*	1.87	42.83	37.08	37.47	24.82
8	0.05+	10.99	1.41+	0.19	22.30	39.62	32.28	29.26
9	22.52	24	13.1	15.44*	31.26	56.68*	38.01	41.98
10	36.77*	1.83	7.11	14.21	46.71	30.00	30.96	34.12
11	24.94	7.99	7.24	0.06	34.48	32.46	38.42	21.34

Auxiliary traits of +major and *minor importance in the prediction of PT1 Scenario 1 represents the first four control loci, scenario 2 represents the last four control loci, scenario 3 represents the first two and last two control loci, and scenario 4 represents the central control loci (excluding the first and last two loci).

Given the great complexity of interpreting the MLP network to quantify the relative importance of traits, an alternative is to use Garson’s algorithm (1991) modified by Goh [34]. This algorithm partitions the ANN connection weights to determine the relative importance of each input trait. The weights associated with the neurons in an ANN are partially analogous to the coefficients in a generalized linear model [51]. The combined effects of the weights represent the relative importance of the predictors for predicting the response variable. The weights correspond to the relative influence of the information that is processed in the network such that the input variables that are not relevant are suppressed by the weights.

The method proposed by Garson (1991), modified by Goh [34], identifies the relative importance of explanatory variables toward specific response variables in a supervised neural network. The relative importance (or strength of association) of a specific explanatory variable to a specific response variable can be determined by identifying all the weighted connections between the nodes of interest. This algorithm has the ability to deal with degrees of association and multicollinearity between explanatory characteristics and has been shown to be efficient in quantifying the importance of phenotypic characteristics in studies with known genetic structures.

The percent relative contributions of the 10 phenotypic traits relative to PT1 in the four scenarios estimated by this method are listed in Table 7. In all scenarios, the relative contributions of PT5 and PT2 in predicting PT1 were quantified as major and minor, respectively. The result for PT2 was expected because of the lack of shared LGs controlling PT1. Thus, PT5 presented the highest number of major pleiotropic markers, in addition to the minor markers, but with major heritability.

**Table 7:** Percentage of relative contribution using the Garson’s algorithm (1991) modified by Goh (1995) for 10 phenotypic traits (PTs) relative to PT1 in the four scenarios.

PT	Scenarios			
	1	2	3	4
2	6.12*	5.24*	8.28*	8.77*
3	9.26	8.65	10.63	9.27
4	7.89	11.13	9.47	10.26
5	13.11+	12.04+	11.41+	12.73+
6	9.47	10.16	9.00	9.11
7	9.87	10.49	8.98	9.79
8	11.14	11.96	10.64	10.16
9	10.81	9.85	11.03	10.35
10	10.77	9.96	10.43	11.33
11	11.55	10.53	11.12	11.21

Auxiliary traits of +major and *minor importance in the prediction of PT1 Scenario 1 represents the first four control loci, scenario 2 represents the last four control loci, scenario 3 represents the first two and last two control loci, and scenario 4 represents the central control loci (excluding the first and last two loci).

ANNs most often exhibit satisfactory performance compared with other machine learning-based predictive algorithms [17]. The MLP network has been widely used in predictive processes [17, 52], and its success has already been demonstrated in several studies, which mathematically showed that networks even with only a single hidden layer with different numbers of neurons work very well [17, 53].

#### RBF

The results of quantification of the importance of phenotypic traits using RBF after the permutation of the traits and assignment of a zero value to the input trait are shown in Table 8.

**Table 8:** Estimation of the coefficient of determination (*R*^2^) for the prediction of phenotypic trait 1 (PT1) using the radial basis function (RBF).

PT	Zero				Permutation			
	1	2	3	4	1	2	3	4
2	37.15*	9.38	27.92	37.99*	47.96	32.27	33.27	37.82
3	12.63	23.66	15.44	25.94	27.35	37.66	24.24	37.01
4	19.39	23.39	21.65	18.91	24.96	24.40	36.66	32.15
5	8.40	8.60+	9.73+	19.91	9.31+	16.19+	14.46+	16.86+
6	32.37	20.21	15.79	30.83	44.45	29.01	33.78	38.95
7	28.17	19.46	33.69	19.94	44.39	40.89*	31.02	33.63
8	29.26	13.03	11.02	9.79+	43.74	38.73	33.11	39.69
9	36.10	36.20*	22.62	28.53	33.47	36.65	39.25	28.79
10	39.41	26.63	32.27	19.12	50.79*	39.77	39.40*	40.38*
11	2.01+	30.15	37.32*	25.23	29.40	40.14	34.84	30.49

Auxiliary traits of +major and *minor importance in the prediction of PT1 Scenario 1 represents the first four control loci, scenario 2 represents the last four control loci, scenario 3 represents the first two and last two control loci, and scenario 4 represents the central control loci (excluding the first and last two loci).

The relative importance of phenotypic traits based on the reduction in *R*^2^ (Table 8), independent of the heritability of each phenotypic trait and LGs, differed across scenarios. The most important traits obtained with the permutation of traits and assignment of a zero value to the input trait were not consistent. As mentioned earlier, permutation was efficient in quantifying the relative contribution of PT5 as a major based on the reduction in *R*^2^ estimate when the information was disturbed (Table 7). This efficiency could be extended to PT3, PT4, and PT5, which shared the same number of markers as PT1 and differed only in terms of heritability. Using this strategy, PT2 was identified as the least important trait.

Radial basis function networks have the ability to learn from the data used in their training and provide a unique solution. They are also comparatively faster than perception-type ANNs [26]. In addition, they have a good ability to handle interactions compared to semi-parametric and linear regressions [28]. Sant’Anna et al. [28] applied RBF in studies using simulated characteristics with 30% and 60% heredity for variable selection. The authors identified greater efficiency in the selection using RBF when the scenario involved epistatic interactions in the gene control of the studied characters. González-Camacho et al. [26] observed that it is possible to improve prediction in non-parametric models when the selection includes markers that are not directly related to the characteristics of interest.

The results obtained corroborate the expectation about the RBF in quantifying and revealing the importance of the characteristics using the strategy of causing disturbances based on the permutations or fixation of the phenotypic values of the input variables.

Our study demonstrates the ability of RNA to quantify the importance of phenotypic characteristics with known genetic structures. Techniques showing the impact of the disruption or disturbance to the information of a given entry on the estimation of the determination coefficient and partitioning of the ANN connection weights have been presented. These techniques were effective in quantifying the true importance of phenotypic characteristics.

### Importance of phenotypic traits by machine learning

The importance of phenotypic traits using machine learning and its refinements (bagging, random forest, and boosting) in four different scenarios are shown in Table 9. The %IMSE values were calculated, with the highest value representing the most important phenotypic trait.

**Table 9:** Average estimate of the relative contribution of explanatory phenotypic traits (PTs) to the prediction of PT1 using machine learning in the four scenarios.

PT	Bagging				Random forest				Boosting			
	1	2	3	4	1	2	3	4	1	2	3	4
2	8.35*	10.63	16.5	13.14	7.32	13.79	15.71	15.94	1.08*	3.20	11.18	11.42
3	20.50	25.36	18.23	12.70	22.51	25.27	18.39	13.81	11.92	19.22	16.58	9.88
4	29.58	14.72	19.22	23.92	28.7	14.69	20.33	24.85	26.69	6.73	13.04	27.57
5	34.13+	40.45+	24.8+	29.36+	33.9+	34.78+	25.56+	26.98+	33.82+	31.31+	23.28+	24.67+
6	10.67	16.48	7.18	9.17	9.98	16.4	7.82*	10.05	3.23	8.89	5.85	5.94
7	12.22	9.45*	12.42	7.75	13.65	8.51*	13.46	8.38	3.89	2.39*	9.35	1.69
8	12.28	11.99	9.34	5.41	12.96	11.89	9.31	6.03	3.64	3.86	3.88	1.68*
9	13.00	14.24	7.55*	11.57	13.63	14.99	8.59	14.11	4.71	7.70	1.82*	6.82
10	4.03	15.59	11.64	4.00*	3.82*	15.92	13.05	5.35*	3.20	10.68	4.96	2.62
11	15.43	11.61	18.6	14.48	15.9	12.62	15.03	16.39	7.82	6.04	10.07	7.70

Auxiliary traits of +major and *minor importance in the prediction of PT1 Scenario 1 represents the first four control loci, scenario 2 represents the last four control loci, scenario 3 represents the first two and last two control loci, and scenario 4 represents the central control loci (excluding the first and last two loci).

PT5 was estimated as the most important phenotypic trait in all machine learning methodologies and in all scenarios. This result is consistent with that of the computational intelligence methods (Tables 6–8). Although machine learning is an efficient tool for quantifying the relative importance of traits, it does not make any assumptions regarding the distribution of explanatory variables and is robust in terms of quantity, redundancy, and environmental influence [19, 54]. In addition, random forest and boosting do not require an inheritance specification model and can account for non-additive effects without increasing the number of covariates in the model or computation time [55]. Random forest and bagging show good predictive performance in practice; they work well for multi-dimensional problems and can be used with multi-class output, categorical predictors, and imbalanced problems [56]. Satisfactory results of variable selection using the random forest algorithm in the presence of correlated predictors have been reported for [56].

The discriminatory power, redundancy, precision, and complexity can influence the indices or statistics used to quantify the importance of auxiliary traits in the prediction of a principal trait. Thus, the selection of a prediction method and index reflecting the true importance of each auxiliary trait is imperative. In the present study, we propose some genetic structures that can allow us to estimate the efficiency of procedures using computational intelligence and machine learning to quantify the relative contribution of phenotypic traits in different scenarios for the implementation of these techniques in other studies. The lack of information on the implementation of computational intelligence and machine learning in breeding programs to predict phenotypic traits is a challenge for breeders. However, with the relevant recent advances in biotechnology and high-throughput phenotyping, more information can be obtained for the identification of genetically superior individuals.

Breeding for desired traits in crops has long been a time-consuming, labor-intensive, and expensive process. Breeders study generations of plants and identify and modify desired genetic traits, as they assess how traits are expressed in offspring [57, 58]. The application of computational intelligence and machine learning to identify optimal suites of observable characteristics (phenotypes) can enable informed decisions and achieve outcomes of great relevance in breeding programs. In addition, these methodologies can help predict genetic traits with the best performance under different agricultural management practices.

Methodologies based on machine learning and computational intelligence do not depend on stochastic information and tend to be more efficient, whereas conventional methodologies depend on the normality of the distribution of phenotypic traits. Moreover, machine learning and computational intelligence methodologies make no assumptions regarding the model and can capture complex factors, such as epistasis and dominance, in prediction models. In machine learning, *a priori* knowledge of prediction is not required if the data produce these effects, and no assumptions are made regarding the distribution of phenotypic values [59]. Machine learning algorithms have the advantage of modeling data in a non-linear and non-parametric manner [60]. Unlike many traditional statistical methods, these algorithms are built with the advantage of dealing with noisy, complex, and heterogeneous data [61–64] reported that machine learning methods are powerful tools for predicting genetic values with epistatic genetic control in traits with different degrees of heritability and different numbers of controlling genes. The results obtained in the present study can be used to select genotypes and test them in the field. Thus, the proposed model can be validated in practice.

## Conclusion

Computational intelligence and machine learning can efficiently predict the relative contributions of auxiliary traits in different scenarios in plant breeding programs. PT5 was identified as the most important predictor of PT1.

## Supporting information

S1 File (RAR)
